# Effects of Health-Related Food Taxes and Subsidies on Mortality from Diet-Related Disease in New Zealand: An Econometric-Epidemiologic Modelling Study

**DOI:** 10.1371/journal.pone.0128477

**Published:** 2015-07-08

**Authors:** Cliona Ni Mhurchu, Helen Eyles, Murat Genc, Peter Scarborough, Mike Rayner, Anja Mizdrak, Kelechi Nnoaham, Tony Blakely

**Affiliations:** 1 National Institute for Health Innovation, University of Auckland, Auckland, New Zealand; 2 Department of Economics, University of Otago, Dunedin, New Zealand; 3 British Heart Foundation Centre on Population Approaches for Non-Communicable Disease Prevention, University of Oxford, Oxford, United Kingdom; 4 Department of Public Health, University of Otago, Wellington, New Zealand; University of Cambridge, UNITED KINGDOM

## Abstract

**Background:**

Health-related food taxes and subsidies may promote healthier diets and reduce mortality. Our aim was to estimate the effects of health-related food taxes and subsidies on deaths prevented or postponed (DPP) in New Zealand.

**Methods:**

A macrosimulation model based on household expenditure data, demand elasticities and population impact fractions for 18 diet-related diseases was used to estimate effects of five tax and subsidy regimens. We used price elasticity values for 24 major commonly consumed food groups in New Zealand, and food expenditure data from national Household Economic Surveys. Changes in mortality from cardiovascular disease, cancer, diabetes and other diet-related diseases were estimated.

**Findings:**

A 20% subsidy on fruit and vegetables would result in 560 (95% uncertainty interval, 400 to 700) DPP each year (1.9% annual all-cause mortality). A 20% tax on major dietary sources of saturated fat would result in 1,500 (950 to 2,100) DPP (5.0%), and a 20% tax on major dietary sources of sodium would result in 2,000 (1300 to 2,700) DPP (6.8%). Combining taxes on saturated fat and sodium with a fruit and vegetable subsidy would result in 2,400 (1,800 to 3,000) DPP (8.1% mortality annually). A tax on major dietary sources of greenhouse gas emissions would generate 1,200 (750 to 1,700) DPP annually (4.0%). Effects were similar or greater for Maori and low-income households in relative terms.

**Conclusions:**

Health-related food taxes and subsidies could improve diets and reduce mortality from diet-related disease in New Zealand. Our study adds to the growing evidence base suggesting food pricing policies should improve population health and reduce inequalities, but there is still much work to be done to improve estimation of health impacts.

## Introduction

Poor diets account for a substantial proportion of death and disability worldwide, with the major dietary risks being low fruit intake and high salt [1]. The global burden of disease due to diet-related physiological risk factors such as high blood pressure, high body-mass index (overweight and obesity), and high blood cholesterol is also considerable [1]. Improving diets and reducing salt intakes were identified as priority areas for international action at the United Nations High-Level Meeting on diet-related diseases in 2011 [2].

In New Zealand, almost one third of adults (31%) and one in 10 children (11%) are obese [3], and poor diets and obesity combined account for more death and disability than tobacco [4]. Obesity rates are highest amongst Pacific (68%) and Māori (48%) adults, whilst those living in the most deprived areas are 1.5 times more likely to be obese than those living in the least deprived areas [3].

Health-related food taxes and subsidies, where the price of unhealthy foods is increased and/or that of healthy foods is decreased, are a potential means to promote healthier diets [5–9]. In 2011, Denmark introduced a saturated fat tax (since repealed); Hungary implemented a tax on foods high in sugar, sodium or caffeine; France applied a tax on soft drinks; and Finland a tax on confectionery [7]. This year, Mexico, which has the second highest rate of obesity in the OECD, implemented a law imposing taxes on sugar-sweetened drinks and foods high in saturated fat, sugar and sodium [10]. Most recently, Berkeley became the first city in the United States to tax sugar-sweetened beverages [11].

Systematic reviews suggest that these types of health-related food taxes and subsidies would be associated with beneficial dietary changes, and show potential for improved health [12–14]. Natural experiments also indicate that taxes on saturated fat and sugar-sweetened beverages are effective in reducing consumption of targeted foods [15,16]. However important gaps in the existing evidence base hinder their adoption and implementation in many countries. Gaps include the effects of compensatory purchasing of non-targeted food items (cross-price elasticity effects); impact on different socioeconomic groups; and effects on long-term health and mortality [13]. Appropriate price elasticities (PE), required to quantify proportional change in consumption for a given change in price (as well as cross-price elasticities to quantify how a price change to food ‘A’ impacts on consumption of food ‘B’), are often lacking too.

Our objective was to estimate the effects of health-related food taxes and subsidies on mortality from diet-related diseases in New Zealand. We specifically aimed to incorporate any impact of compensatory food purchasing (via inclusion of both own- and cross-price PE in models); to use New Zealand-specific PE estimates because different PE values may lead to different modelling results between countries; to incorporate uncertainty around both PE estimates and epidemiological parameters in the model; and evaluate effects by income and ethnicity.

## Materials and Methods

Taxation and subsidy scenarios (Table 1) were selected for inclusion in our models if they met at least one of the following criteria during the study design period (2011): (1) recently considered for implementation in New Zealand (subsidy on fruit and vegetables); (2) recently implemented in another high income country (saturated fat tax); or (3) topical in international public health literature (sodium tax and greenhouse gas tax). We initially included a soft drink tax scenario, but the cross price elasticities generated were implausible Specifically, the cross-PE for carbonated soft drinks to energy drinks was +2.73 (S1 Table), implying that a 10% increase in the price of carbonated drinks would result in a 27% increase in energy drink consumption. Usually cross-PEs are much closer to zero (both theoretically, and empirically as seen in our New Zealand estimates for most other cross-PEs (S1 Table)). We therefore considered our PE data invalid for estimating soft drink taxes.

**Table 1:** Health-related taxation and subsidy regimens modelled.

Health-related food tax/subsidy	Magnitude and format
Fruit and vegetable subsidy	20% flat rate subsidy on total cost of fruit and vegetables (dried, canned, frozen, and fresh)
Saturated fat tax	20% tax on total cost of major food contributors to saturated fat intakes [17] (butter; cakes and biscuits; cheese and cream; pastry cook products; beef, lamb and hogget; poultry; and prepared, preserved, and processed meat)
Sodium tax	20% tax on total cost of major food contributors to sodium intakes [17] (bread and breakfast cereals; prepared, preserved and processed meat; sauces and condiments; beef, lamb, and hogget; poultry; and takeaway foods and beverages)
Combination of taxes and subsidy	20% subsidy on total cost of fruit and vegetables + 20% tax on major food group contributors to saturated fat and sodium intakes (maximum 20% tax was applied to foods high in both saturated fat and sodium)
Greenhouse gas tax	20% tax on total cost of major food group contributors to carbon emissions [59] (milk, yoghurt and eggs; cheese and cream; butter; ice cream; beef, lamb and hogget; pork; prepared, preserved and processed meat; and poultry)

We modelled flat rate taxes of 20% applied to the retail price of major food group contributors to targeted nutrients (Table 1), e.g. the saturated fat tax was applied to food groups that contributed 5% or more to saturated fat intakes in New Zealand (butter; cakes and biscuits; cheese and cream; pastry cook products; beef, lamb and hogget; poultry; and prepared, preserved, and processed meat) [17]. A 20% subsidy on fruit and vegetables was also modelled.

Selection of food taxes and subsidies for modelling was constrained to some extent by the expenditure data used to define baseline food purchasing habits. Within the national household food expenditure datasets, nutritionally diverse foods are often aggregated into a single category, e.g. all milk and dairy products (both lower and higher fat options) were combined in one category. This meant the saturated fat tax model was applied to both reduced-fat and regular dairy products, rather than the more ideal scenario of full-fat dairy products only.

### Effects of price changes on food and nutrient purchases

**Fig 1:**
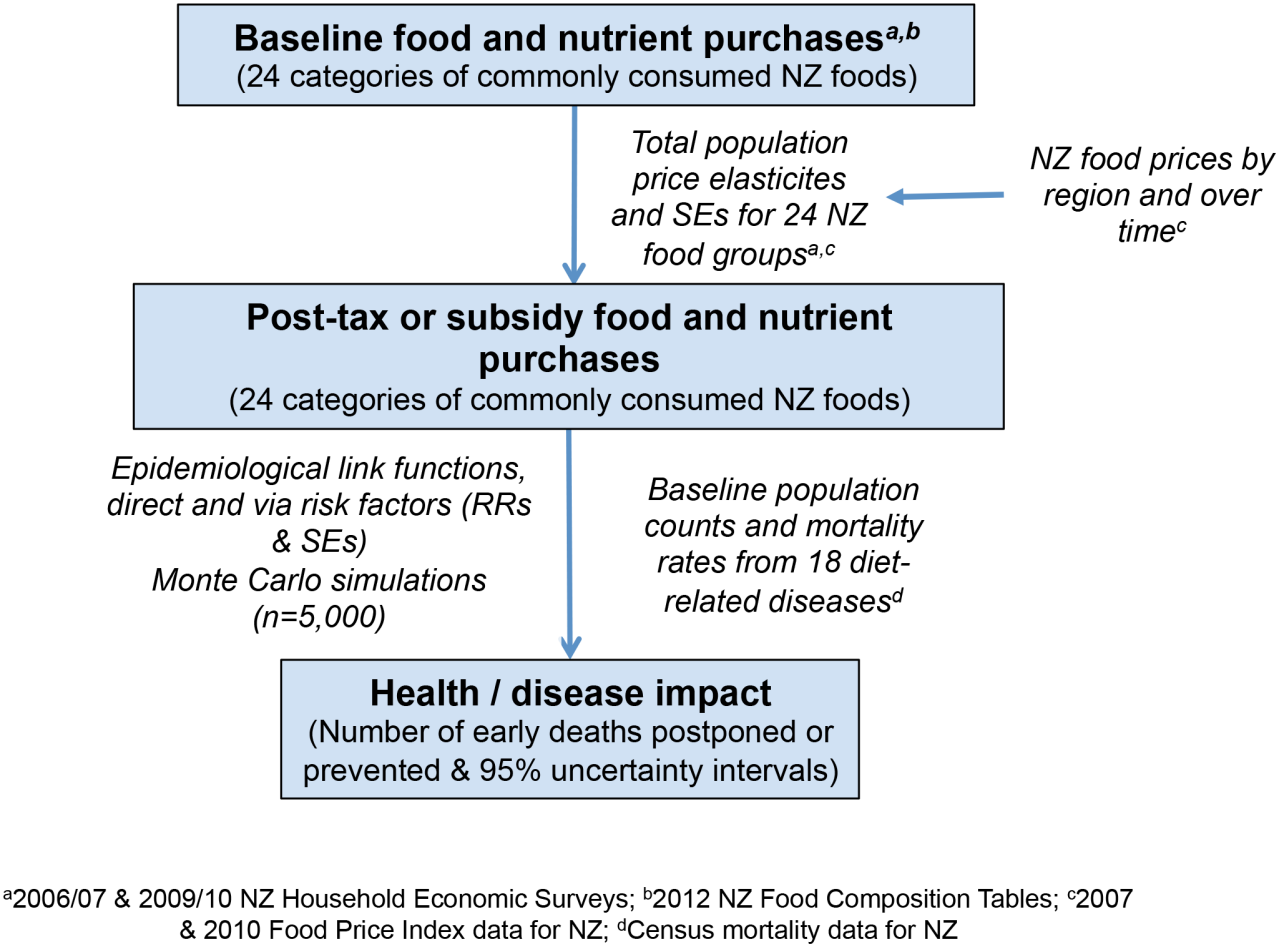
Data sources used as inputs to model.

The various data sources for the model are outlined in Fig 1. Baseline data on food purchasing and expenditure were derived from the New Zealand Household Economic Surveys (HES) for 2006/07 and 2009/10 (n = 6,028 households in total) [18,19]. Food items in the HES datasets were linked with nutrient data from the New Zealand Food Composition Tables [20] to derive the nutritional composition of the baseline diet. Households were classified as Māori or non-Māori based on prioritised ethnicity of the reference household member, a system commonly used in New Zealand.[21] Where more than one ethnicity was recorded, households were classified as Māori if they identified as Māori, regardless of the order of ethnic groups listed.[21] All other households were classified as non-Māori. Households were classified into income tertiles based on total recorded annual (non-equivalised) household income: <$26,109; $26,109 to $43,016, and ≥$43,016.

We used PE estimates to simulate changes in total food purchasing if selected foods were taxed or subsidised. PEs were estimated for 24 New Zealand food groups using data from HES 2007/08 and 2009/10 [18,19] that were manually linked to Food Price Index data for 2007 and 2010 [22]. Own-PE and cross-PE estimates were derived by estimating an Almost Ideal Demand System model as described in [23] and applying the Shonkwiler and Yen procedure [24] to deal with censoring (i.e., existence of zero expenditures). The procedure consists of two steps where maximum likelihood probit predictions of the probability and marginal change in probability of observing nonzero observations are obtained for each food group in the first step. The regressors in each budget share equation are then weighted by the predicted probability of purchasing the good, and the equation is augmented by the corresponding marginal probability before the demand system is estimated. The full PE matrix used in current models is available as a supporting information file (S1 Table).

To maximise model stability, each of the five taxation/subsidy models used own-PE and cross-PE values for the food groups targeted in each specific scenario and all theoretical substitutes and complements [25] instead of the full PE matrix for all 24 food groups (552 PE values) (Table 2). In effect, we assumed that the cross-PE between two food groups that are unrelated was zero, thereby reducing random noise in the modelled output—a process that is implicitly applied when selecting the demand structure for modelling single food tax scenarios [26], and has been explicitly applied in other studies that modelled multiple tax scenarios [27]. The output estimated new dietary intakes in response to the five different tax and subsidy regimens.

**Table 2:** Food group price elasticity values included in taxation and subsidy models.

Health-related food tax/subsidy	Own- and cross-price elasticity values included in specific models*
Fruit and vegetable subsidy	Fruit; Vegetables; Cakes and biscuits; Chocolate, confectionary and snacks; Ice cream; Beef, lamb, and hogget; Poultry; Pork; Prepared, preserved and processed meat; Fish and seafood; Pasta and other cereal products
Saturated fat tax	Butter; Cakes and biscuits; Cheese and cream; Pastry cook products; Beef, lamb and hogget; Poultry; Prepared, preserved and processed meat; Pork; Fish and seafood; Pasta and other cereal products; Sauces, sugar & condiments; Margarine and edible oil; Chocolate, confectionary and snacks
Sodium tax	Bread & breakfast cereals; Prepared, preserved, and processed meat; Sauces, sugar & condiments; Beef, Lamb, and hogget; Poultry; Ready to eat food; Pork; Fish and seafood; Cakes and biscuits; Pasta, and other cereal products; Milk, yoghurt, and eggs; Cheese and cream; Butter, Margarine and edible oil
Combination scenario	All own- and cross-PE values used in previous four models
Greenhouse gas tax	Milk, yoghurt, and eggs; Cheese and cream; Butter; Ice cream; Beef, Lamb, and hogget; Pork; Prepared, preserved, and processed meat; Poultry; Fish and seafood; Pastry cook products; Pasta, and other cereal products; Milk, yoghurt, and eggs; Cheese and cream; Sauces, sugar & condiments; Ice cream

* Includes all own-price elasticities in addition to theoretical complements and substitutes

### Effects of changes in food and nutrient purchases on nutrition and mortality

We modelled changes in nutritional risk factors on mortality from diet-related diseases using the PRIME comparative risk assessment macrosimulation model [28]. PRIME models consumption of foods (fruit and vegetables) and nutrients (dietary fibre, fats, dietary cholesterol, sodium, energy) through to biological risk factors for ill health (blood pressure, total serum cholesterol, obesity), and thence to nutrition-related mortality (cardiovascular disease, diabetes, diet-related cancers and other diet-related NCDs) [28]. Meta-analyses are used to parameterise changes in nutritional risk factors and mortality as a result of changes in population foods and nutrients [29–36]. PRIME estimates the difference in mortality in one single year between the baseline (in this case, 2009) and the counterfactual scenario (also 2009, but a 2009 in which the scenarios modelled have always been in place). If time lag is ignored, then the results can be interpreted as annual deaths prevented or postponed as a result of the scenarios.

95% uncertainty intervals (95% UI) are generated by Monte Carlo simulations (n = 5000) to reflect uncertainty around the input parameters of price elasticities and relative risks. The PRIME model has been used to model the effects of dietary changes on obesity rates and mortality due to chronic disease in the UK [26,37–39], Ireland [40], France [41] and Canada [42].

A number of key assumptions underpinned our modelling analyses. These were: (1) changes in risk associated with changes in more than one food component were combined multiplicatively (wherever possible the relative risks included in the model were mutually adjusted in order to minimise the effect of double counting, although because these are drawn from meta-analyses of the extant literature it was not possible to ensure complete consistency in statistical adjustments); (2) with the exception of obesity, which follows a J-shaped curve, changes in risk followed a log-linear dose-response relationship; (3) relative risks were consistent across income groups and ethnic groups; (4) changes between baseline and counterfactual food and nutrient consumption distributions were made by all individuals within the population equally; (5) the tax/subsidy pass through rate to the consumer was 100%; and (6) counterfactual dietary intakes were rescaled to maintain the same total baseline dietary energy intakes where fruit and vegetable intakes increased (fruit and vegetable subsidy scenario only) to reflect evidence that increased fruit and vegetable consumption does not increase energy intakes [43].

Population demographics were obtained from the 2006 New Zealand Census of Population and Dwellings [44]. Population disease-specific mortality rates by age, sex, and ethnic group were obtained from national mortality data for 2009 [45], with further disaggregation by income using rate ratios from linked census-mortality data [46,47], and coded using International Classification of Disease (ICD) codes.

### Primary and secondary analyses

Own-PE and cross-PE values derived for ethnic and income sub-groups proved to be unstable and highly variable so the impact of health-related food taxes and subsidies on consumption was estimated in two ways. The primary analysis applied the selected theoretical own- and cross-PEs for the total New Zealand population to all income and ethnic sub-groups. Differences in results for these subgroups is therefore due to differences in baseline diets and mortality between groups. A sensitivity analysis included the same theoretical own- and cross-PEs in the models but increased the own-PE values by an average of 30% for low-income households and 26% for Māori households to reflect known significant differences in New Zealand consumer demand by income and ethnicity [23].

DPP in tables are presented as unrounded estimates, however in the text estimates are reported to the nearest two meaningful digits to reinforce the uncertainty inherent in the modelling.

## Results

The baseline New Zealand population diet estimated from household food expenditure data was low in fruit (136g/day), vegetables (201g/day) and fibre (16.4g/day), and high in saturated fat (14.7% energy) and sodium (2,860 mg/day) compared with national dietary targets to reduce chronic disease risk [48]. There were minimal differences in baseline diet by household income tertile. However, Māori diets were unusually low in absolute intakes of energy, fruit, vegetable, fibre and salt (20–40% lower than those of non- Māori), which possibly reflected the relatively small number of Māori survey participants (n = 578, 10%) and/or reporting bias. Therefore Māori diets were recalibrated using ratio of Māori: non-Māori intakes derived from the most recent National Nutrition Survey [17]. Recalibration increased Māori energy intakes by 37%, fruit and vegetable intakes by approximately 60%, and sodium by 26%. Following recalibration there were no important differences in baseline diet by ethnicity, which is broadly consistent with National Nutrition Survey findings [17].

### Changes in food and nutrient purchases

Our model predicts that a 20% subsidy of price of fruit and vegetables would increase total population fruit and vegetable purchases by approximately 12% (95% UI 9.6% to 13.6%) and 18% (95% UI 16.5% to 18.9%) respectively, whilst saturated fat purchases would decrease by about 1% and sodium purchases would decrease by approximately 0.6% (Table 3). Taxes on major dietary sources of saturated fat and sodium would reduce daily population energy purchases by approximately 5% (111 kCal/day) and 7% (170 kCal/day) respectively. A saturated fat tax would reduce saturated fat and sodium purchases by approximately 6% each, whilst a sodium tax would reduce sodium intakes by approximately 11% but would result in a 2% increase in saturated fat purchases due to positive cross-PEs between pork and products high in sodium. Both taxation scenarios would lead to small decreases in vegetable purchases (approximately 2–3%), and a saturated fat tax would also lead to a 3% decrease in fruit purchases (due to positive cross-PEs between foods that contribute to saturated fat intake in New Zealand (e.g. cheese and cream; chocolate confectionary and snacks; ice-cream), and fruit. Combining taxes on foods high in saturated fat and sodium with a 20% subsidy on fruit and vegetables would produce the most positive effects on consumer food purchases and diets. A tax on major dietary sources of greenhouse gas emissions would have favourable effects on population purchases of energy, saturated fat and sodium, but would result in a concomitant 2.5% decrease in vegetable purchases.

**Table 3:** Changes in food and nutrient purchases overall and by ethnicity and income.

	% change in total energy		% change in saturated fat		% change in sodium		% change in fruit		% change in vegetables	
	Mean	95% UI	Mean	95% UI	Mean	95% UI	Mean	95% UI	Mean	95% UI
**Fruit and vegetable subsidy** *										
Total population (n = 5,991)	0.00	(0.00, 0.00)	-0.98	(-1.12, -0.83)	-0.61	(-0.77, -0.45)	11.66	(9.58, 13.64)	17.68	(16.47, 18.93)
Māori (n = 569)	0.00	(0.00, 0.00)	-0.80	(-0.93, -0.68)	-0.53	(-0.66, -0.40)	11.66	(9.60, 13.68)	17.68	(16.44, 18.92)
Non-Māori (n = 5,420)	0.00	(0.00, 0.00)	-0.99	(-1.15, -0.84)	-0.61	(-0.78, -0.45)	11.66	(9.61, 13.70)	17.68	(16.43, 18.92)
Low income (n = 1,077)	0.00	(0.00, 0.00)	-1.05	(-1.20, -0.89)	-0.63	(-0.81, -0.45)	11.66	(9.60, 13.70)	17.68	(16.48, 18.91)
Middle income (n = 1,045)	0.00	(0.00, 0.00)	-1.00	(-1.14, -0.86)	-0.59	(-0.74, -0.43)	11.66	(9.67, 13.62)	17.68	(16.43, 18.91)
High income (n = 3,869)	0.00	(0.00, 0.00)	-0.96	(-1.11, -0.81)	-0.61	(-0.77, -0.44)	11.66	(9.65, 13.70)	17.68	(16.46, 18.94)
**Saturated fat tax**										
Total population (n = 5,991)	-4.69	(-5.36, -4.01)	-5.83	(-7.12, -4.56)	-6.02	(-6.77, -5.25)	-3.36	(-5.43, -1.26)	-2.79	(-4.77, -0.79)
Māori (n = 569)	-4.86	(-5.54, -4.20)	-5.96	(-7.37, -4.60)	-5.94	(-6.65, -5.21)	-3.36	(-5.40, -1.25)	-2.79	(-4.84, -0.75)
Non-Māori (n = 5,420)	-4.67	(-5.36, -3.97)	-5.81	(-7.04, -4.58)	-6.02	(-6.79, -5.26)	-3.36	(-5.48, -1.28)	-2.79	(-4.81, -0.76)
Low income (n = 1,077)	-4.66	(-5.45, -3.88)	-6.18	(-7.73, -4.60)	-6.33	(-7.16, -5.50)	-3.36	(-5.46, -1.30)	-2.79	(-4.84, -0.76)
Middle income (n = 1,045)	-4.73	(-5.47, -3.98)	-6.10	(-7.62, -4.60)	-6.31	(-7.08, -5.50)	-3.36	(-5.50, -1.34)	-2.79	(-4.84, -0.79)
High income (n = 3,869)	-4.68	(-5.36, -3.98)	-5.73	(-6.86, -4.57)	-5.92	(-6.66, -5.14)	-3.36	(-5.42, -1.27)	-2.79	(-4.77, -0.78)
**Sodium tax**										
Total population (n = 5,991)	-7.17	(-7.63, -6.70)	2.19	(1.53, 2.87)	-10.77	(-11.35, -10.16)	0.00	(0.00, 0.00)	-1.90	(-3.38, -0.38)
Māori (n = 569)	-7.22	(-7.71, -6.74)	2.18	(1.46, 2.91)	-10.44	(-11.07, -9.85)	0.00	(0.00, 0.00)	-1.90	(-3.42, -0.40)
Non-Māori (n = 5,420)	-7.17	(-7.64, -6.69)	2.19	(1.50, 2.84)	-10.80	(-11.42, -10.19)	0.00	(0.00, 0.00)	-1.90	(-3.45, -0.40)
Low income (n = 1,077)	-7.07	(-7.58, -6.58)	2.81	(2.00, 3.62)	-10.43	(-11.05, -9.79)	0.00	(0.00, 0.00)	-1.90	(-3.42, -0.38)
Middle income (n = 1,045)	-7.24	(-7.73, -6.76)	2.88	(2.10, 3.67)	-10.62	(-11.25, -10.00)	0.00	(0.00, 0.00)	-1.90	(-3.38, -0.34)
High income (n = 3,869)	-7.17	(-7.64, -6.72)	1.97	(1.34, 2.60)	-10.84	(-11.45, -10.25)	0.00	(0.00, 0.00)	-1.90	(-3.43, -0.42)
**Combination scenario** **										
Total population (n = 5,991)	-6.15	(-6.89, -5.38)	-3.64	(-4.92, -2.36)	-10.79	(-11.58, -9.94)	11.66	(9.62, 13.62)	14.89	(12.52, 17.25)
Māori (n = 569)	-6.73	(-7.46, -5.98)	-3.63	(-5.06, -2.21)	-10.54	(-11.37, -9.73)	11.66	(9.68, 13.66)	14.89	(12.49, 17.20)
Non-Māori (n = 5,420)	-6.09	(-6.84, -5.33)	-3.64	(-4.87, -2.40)	-10.82	(-11.66, -9.96)	11.66	(9.71, 13.73)	14.89	(12.54, 17.23)
Low income (n = 1,077)	-5.89	(-6.73, -5.05)	-3.78	(-5.31, -2.24)	-10.48	(-11.34, -9.58)	11.66	(9.65, 13.66)	14.89	(12.45, 17.33)
Middle income (n = 1,045)	-6.20	(-7.03, -5.37)	-3.52	(-5.01, -2.04)	-10.68	(-11.57, -9.80)	11.66	(9.65, 13.68)	14.89	(12.49, 17.26)
High income (n = 3,869)	-6.17	(-6.93, -5.40)	-3.65	(-4.83, -2.48)	-10.85	(-11.66, -10.01)	11.66	(9.64, 13.66)	14.89	(12.49, 17.25)
**Greenhouse gas emissions tax**										
Total population (n = 5,991)	-3.58	(-4.20, -2.96)	-3.72	(-4.72, -2.69)	-3.08	(-3.73, -2.42)	0.00	(0.00, 0.00)	-2.53	(-5.04, 0.03)
Māori (n = 569)	-3.73	(-4.32, -3.11)	-3.79	(-4.95, -2.66)	-3.02	(-3.67, -2.36)	0.00	(0.00, 0.00)	-2.53	(-5.04, 0.11)
Non-Māori (n = 5,420)	-3.56	(-4.20, -2.91)	-3.71	(-4.72, -2.69)	-3.08	(-3.75, -2.42)	0.00	(0.00, 0.00)	-2.53	(-5.03, 0.01)
Low income (n = 1,077)	-3.73	(-4.43, -3.03)	-4.15	(-5.46, -2.82)	-3.40	(-4.12, -2.68)	0.00	(0.00, 0.00)	-2.53	(-5.09, 0.02)
Middle income (n = 1,045)	-3.73	(-4.38, -3.03)	-3.98	(-5.21, -2.76)	-3.29	(-3.97, -2.58)	0.00	(0.00, 0.00)	-2.53	(-5.12, 0.01)
High income (n = 3,869)	-3.53	(-4.13, -2.91)	-3.61	(-4.55, -2.64)	-2.99	(-3.66, -2.32)	0.00	(0.00, 0.00)	-2.53	(-5.08, 0.04)

*Energy from increased fruit and vegetable purchases not included in model i.e. energy was kept constant between baseline and counterfactual diets [43].

** Energy from increased fruit and vegetable purchases included in model as could not be disaggregated from changes in purchases of energy from other scenarios

### Changes in health outcomes

**Fig 2:**
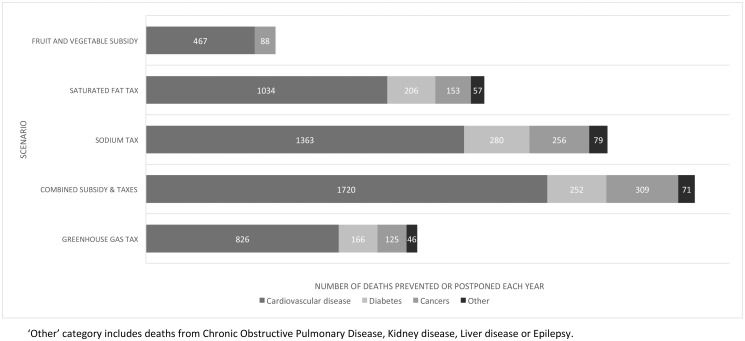
Total number of deaths prevented or postponed in New Zealand each year by five taxation and subsidy scenarios.

All five scenarios modelled would avert or postpone deaths, with estimates ranging from 560 lives saved every year in New Zealand (95% UI 400 to 700) for a fruit and vegetable subsidy (assuming no change in energy consumption), to 2,400 lives saved (95% UI 1,800 to 3,000) with a combination of taxes on foods high in saturated fat and sodium and a subsidy on fruit and vegetables (Table 4). For all scenarios modelled, most mortality averted (70–85%) would be from deaths due to cardiovascular disease (Fig 2). For the fruit and vegetable subsidy scenario, 84% of DPP would be from cardiovascular disease and the remainder would be from cancers. For all other scenarios, 69–73% of DPP would be from cardiovascular disease, 11–14% from diabetes, 11–13% from cancers, and 3–4% from other causes (chronic obstructive pulmonary disease, kidney disease, liver disease or epilepsy).

**Table 4:** Changes in mortality from diet-related diseases, overall and by ethnicity and income.

	Number of CVD deaths averted (95% uncertainty interval)	Number of diabetes deaths averted (95% uncertainty interval)	Number of diet-related cancer deaths averted (95% uncertainty interval)	Total number of early deaths averted (95% uncertainty interval)	% annual deaths
**Fruit and vegetable subsidy** *					
Total population (n = 5,991)	467 (321, 612)	0 (0, 0)	88 (55, 116)	555 (402, 702)	1.9
Māori (n = 569)	39 (27, 51)	0 (0, 0)	9 (6, 13)	48 (36, 61)	1.6
Non-Māori (n = 5,420)	446 (303, 586)	0 (0, 0)	82 (51, 110)	528 (382, 667)	2.0
Low income (n = 1,077)	304 (193, 414)	0 (0, 0)	10 (4, 16)	314 (203, 424)	3.4
Middle income (n = 1,045)	87 (56, 119)	0 (0, 0)	3 (1, 5)	91 (59, 123)	2.9
High income (n = 3,869)	69 (46, 92)	0 (0, 0)	3 (1, 5)	72 (48, 95)	3.3
**Saturated fat tax**					
Total population (n = 5,991)	1034 (552, 1622)	206 (153, 256)	153 (114, 193)	1451 (948, 2051)	5.0
Māori (n = 569)	102 (64, 137)	51 (40, 61)	11 (7, 14)	169 (127, 208)	5.6
Non-Māori (n = 5,420)	966 (501, 1527)	160 (118, 199)	156 (118, 196)	1336 (857, 1914)	5.1
Low income (n = 1,077)	735 (478, 1047)	81 (61, 101)	38 (25, 51)	854 (592, 1173)	9.2
Middle income (n = 1,045)	242 (172, 324)	24 (18, 30)	12 (8, 17)	278 (206, 363)	8.9
High income (n = 3,869)	173 (125, 230)	20 (15, 25)	10 (6, 15)	203 (154, 263)	9.2
**Sodium tax**					
Total population (n = 5,991)	1363 (683, 2,116)	280 (206, 336)	256 (205, 304)	1977 (1289, 2745)	6.8
Māori (n = 569)	127 (74, 173)	70 (58, 81)	19 (14, 24)	225 (168, 272)	7.4
Non-Māori (n = 5,420)	1,281 (648, 1,973)	215 (153, 263)	258 (211, 305)	1,829 (1,180, 2,534)	7.0
Low income (n = 1,077)	1,021 (668, 1,406)	111 (84, 133)	61 (44, 78)	1,193 (833, 1,582)	12.8
Middle income (n = 1,045)	334 (243, 441)	33 (25, 40)	20 (14, 27)	388 (295, 496)	12.4
High income (n = 3,869)	236 (170, 305)	28 (21, 33)	17 (10, 24)	281 (213, 351)	12.7
**Combination scenario**					
Total population (n = 5,991)	1720 (1,150, 2,363)	252 (187, 309)	309 (253, 362)	2352 (1755, 2996)	8.1
Māori (n = 569)	164 (117, 205)	66 (54, 78)	27 (22, 33)	265 (215, 310)	8.8
Non-Māori (n = 5,420)	1,615 (1,099, 2,236)	193 (140, 239)	303 (250, 354)	2,178 (1,640, 2,811)	8.3
Low income (n = 1,077)	1,212 (910, 1,548)	97 (74, 119)	63 (46, 80)	1,372 (1,063, 1,712)	14.8
Middle income (n = 1,045)	391 (308, 485)	30 (23, 37)	21 (15, 28)	443 (358, 538)	14.2
High income (n = 3,869)	284 (227, 350)	25 (19, 31)	19 (12, 25)	328 (269, 394)	14.9
**Greenhouse gas emissions tax**					
Total population (n = 5,991)	826 (430, 1,348)	166 (124, 210)	125 (93, 158)	1163 (749, 1696)	4.0
Māori (n = 569)	79 (50, 109)	40 (32, 49)	9 (7, 12)	134 (99, 167)	4.4
Non-Māori (n = 5,420)	774 (416, 1,240)	129 (94, 164)	127 (95, 160)	1,073 (699, 1,551)	4.1
Low income (n = 1,077)	616 (400, 891)	68 (50, 86)	32 (22, 43)	715 (494, 994)	7.7
Middle income (n = 1,045)	197 (139, 268)	20 (15, 25)	10 (7, 14)	227 (165, 300)	7.3
High income (n = 3,869)	136 (95, 186)	16 (12, 20)	8 (5, 12)	160 (118, 212)	7.2

*Energy from increased fruit and vegetable purchases not included in model i.e. energy was kept constant between baseline and counterfactual diets [43]. DPP for this scenario are greater as a result: 555 (95% uncertainty interval 402, 702) versus 106 (-208, 360) if energy was allowed to increase.

Results are reported in tables unrounded. However, in the text we present results to two meaningful digits only—consistent with, and reinforcing, the inherent uncertainty in these estimates.

### Equity effects

The primary analysis showed no clear gradient of effects of health-related food taxes and subsidies by income (Table 4), other than the GHGe scenario where estimates suggest low-income New Zealanders could gain more than high-income New Zealanders (7.7% of annual deaths amongst low-income households averted versus 7.2% amongst high-income households). All scenarios modelled would benefit Māori more than non-Māori in *relative* terms except the fruit and vegetable subsidy, which would favour non-Māori (2.0% non-Māori annual deaths averted compared with 1.6% for Māori; Table 4), although this would still be a greater *absolute* gain in DPP for Māori per capita by age due to their higher background mortality rates.

The primary analysis used the same price elasticity values for all income and ethnic groups (due to instability of the income- and ethnic-specific PE estimates), which is likely to have under-estimated income and ethnic sub-group effects given that lower-income and Māori consumers exhibit greater sensitivity to changes in the price of most foods [23]. International data also support the premise that changes in food prices have larger effects on demand in low-income countries and households [49,50]. Therefore, we undertook a sensitivity analysis where we increased the own-PE values by an average of 30% for low-income households and 26% for Māori households, consistent with average differences across all food groups [23]. On this assumption, the sensitivity analysis indicates that all pricing scenarios modelled would generate relatively (and more so in absolute terms per capita by age) more DPP amongst Māori and low-income New Zealanders than the total population (Table 5).

**Table 5:** Sensitivity analysis: Changes in total mortality when own-price elasticity values are increased for Māori and low income groups.

	Primary analysis		Sensitivity analysis*	
	Total number of early deaths averted	% annual deaths	Total number of early deaths averted	% annual deaths
**Fruit and vegetable subsidy**				
Total population (n = 5,991)	555	1.9	-	-
Māori (n = 569)	48	1.6	61	2.0
Low income (n = 1,077)	314	3.4	405	4.4
**Saturated fat tax**				
Total population (n = 5,991)	1451	5.0	-	-
Māori (n = 569)	169	5.6	209	6.9
Low income (n = 1,077)	854	9.2	1082	11.7
**Sodium tax**				
Total population (n = 5,991)	1977	6.8	-	-
Māori (n = 569)	225	7.4	267	8.8
Low income (n = 1,077)	1,193	12.8	1411	15.2
**Combination scenario**				
Total population (n = 5,991)	2352	8.1	-	-
Māori (n = 569)	265	8.8	320	10.6
Low income (n = 1,077)	1,372	14.8	1692	18.2
**Greenhouse gas emissions tax**				
Total population (n = 5,991)	1163	4.0	-	-
Māori (n = 569)	134	4.4	168	5.6
Low income (n = 1,077)	715	7.7	920	9.9

* Own-PE values were increased by an average of 30% (1.30) for low-income households and 26% (1.26) for Māori households.

Uncertainty intervals not presented, but not uncertainty still exists consistent with intervals in Table 4.

## Discussion

We modelled the potential effects of health-related food taxes and subsidies on mortality from diet-related non-communicable disease in New Zealand and found that taxes on unhealthy foods, subsidies on healthy foods, and their combination could prevent or postpone substantial numbers of deaths. Effects would be similar or greater for Māori and low-income households in relative terms, and likely greater for Māori and low-income households in absolute terms (DPP per capita within age groups).

### Strengths and weaknesses of the study

Our study has several strengths. We used the most recent New Zealand-specific food price elasticity and food expenditure data; we considered substitution to other foods; population disease-specific mortality rates were stratified by age, sex, income and ethnic group; and baseline diets were stratified by income and ethnicity. The macrosimulation model employed has been used previously to undertake similar analyses for the UK [26,37–39], Ireland [40], France [41] and Canada [42], and the relative risks to parameterise changes in nutritional risk factors and mortality as a result of changes in diet were sourced from high quality meta-analyses [29–36]. Our uncertainty intervals also reflect uncertainty around both PE estimates and the relationship between changes in dietary intake and risk of non-communicable disease.

However, there were some data limitations and necessary assumptions we had to navigate in order to address our objectives. Generation of accurate food PEs is challenging because it requires health policy-relevant groupings of food, accurate price and consumption data, and sufficient variation in price (over time, between regions). The PEs we derived for our models are the best available national estimates but were obtained by linking the economic survey dataset to another dataset, the Food Price Index [23]. They also emerged from a relatively small sample (n = 6,028 households) over a short time period (2006–2010), which led to some unreliable values.

Previously we derived and published PEs by ethnic and socioeconomic group [23], but because of very small numbers in some sub-groups these proved to be unstable and highly variable so we were obliged to use total population PE values in our current income and ethnic subgroup models. However, we undertook a sensitivity analysis where we modified the PE values in line with evidence of significant income- and ethnic-specific effects of pricing [23]. Under this scenario, all of the taxes and subsidies would be pro-equity, averting greater proportions of deaths amongst Māori and low-income New Zealanders than the total population. They would also generate more DPP in absolute terms per capita within age groups for Māori and low-income people due to higher background mortality rates [51].

Furthermore, to maximise model stability we made theoretical assumptions about which cross-price elasticity values to include in the models instead of using the full PE matrix for all 24 food groups. Our assumptions were based on the best available international evidence [25], but it is possible some relevant PEs were not included in the models.

The price elasticities we estimated were ‘conditional’, meaning that the estimation assumed that the total budget share for food did not change. This is a plausible assumption when a single food group undergoes a price change (e.g. a fruit subsidy), but becomes more tenuous when multiple foods undergo price changes, such as with a saturated fat tax. Future research could examine whether allowing for changes in food budget share (e.g. using ‘unconditional’ price elasticities) alters findings. Furthermore, reliable estimates of effects of such pricing policies on household expenditure should allow for change in budget share in line with non-trivial changes in the average price of foods.

It is simple to express health gains in terms of deaths prevented or postponed, or lives saved. However quantification of the health gain in terms of health or quality adjusted life years gained would be better for two key reasons: it captures impact on both quantity and quality of life years gained (a DPP for an 85-year old is very different from one for a 45 year old); and it allows the inclusion of differing background morbidity and mortality rates by sex, age and social group [51].

Additionally, we used the accepted population impact fraction method and calculated deaths postponed in the initial year of policy implementation; it would however take years (e.g. effects of salt reduction on cardiovascular disease) or even decades (e.g. effects of weight loss on cancer), for the full health gain to be realized. Therefore, the results of our study (DPP) should be interpreted as though the tax or subsidy had been in place for several years.

Finally, there is uncertainty regarding potential supply-side responses to taxes or subsidies, including product reformulation to avoid taxes on specific nutrients, use of countervailing marketing campaigns or price promotion strategies (e.g. loss leaders or multi-buy deals) to limit (or amplify) the effects of such pricing policies, and variation in tax/subsidy pass-through rates to the consumer. Testing the sensitivity of results to such supply-side factors is challenging but we were able to do so for tax/subsidy pass-through rates. We assumed that the tax/subsidy pass-through rate to the consumer was 100%. In reality this may not be the case since food manufacturers or retailers might choose to absorb some of the increase in price or increase price beyond the minimum required. We did however assess the sensitivity of our results to plausible variation in the 'base case' tax/subsidy pass-through rate by modelling pass-through rates of +/-20% and, as expected, the effects of taxes/subsidy on mortality were modified accordingly i.e. increased or decreased by approximately 20%. For example, a pass-through rate of 0.8 in the saturated fat tax scenario would result in 1200 DPP each year, versus 1451 DPP with a pass-through rate of 1.0 and 1683 DPP with a pass-through rate of 1.2.

### Comparison with other studies

There is global interest in health-related food taxes and subsidies to improve population diets and health [5,27]. Cost-effectiveness analyses have suggested that taxes on unhealthy foods (e.g. soft drinks, confectionery and snack foods) could be a highly cost-effective population obesity prevention measure [52,53]. Recent modelling studies estimate that a 20% sugar sweetened drink tax would lead to important reductions in prevalence of obesity in the UK [26], Ireland [40] and India [54]. Taxes on palm oil [55] and foods responsible for high greenhouse gas emissions [56] have also been projected to reduce mortality from chronic diseases. Furthermore, in line with our findings, there is emerging evidence that such policies could reduce ethnic- and income-related disparities in obesity and diet-related disease [23,50,57].

All five of the tax and subsidy scenarios we modelled could prevent or postpone deaths in New Zealand. However a similar UK study that modelled the potential health outcomes of four health-related food tax and subsidy regimens for the UK reported that two taxation scenarios (a saturated fat tax and a tax on less healthy foods as defined by a nutrient profiling model—both 17.5% flat rate taxes) would have little effect, or even adverse effects on mortality rates in the case of the saturated fat tax [58]. In contrast, the UK study found that combining a tax on less healthy foods with subsidies on fruit and vegetables would reduce deaths from cardiovascular disease and cancer [58]. The authors suggest that the observed adverse effects of the taxation scenarios on mortality in the UK were due to cross-PEs, for example, between fats and carbohydrates (i.e. as consumption of fats fell consumption of carbohydrates increased, the effects of the latter outweighing the former.) Other potential reasons for observed differences between the UK study and ours are different baseline population diets and use of different PE matrices, suggesting it is necessary to undertake these analyses with population-specific data.

### Interpretation of study findings

Health-related food taxes and subsidies would reduce total population mortality from diet-related disease in New Zealand. Population groups likely to benefit most from such policies are Māori and low-income New Zealanders because their diets are currently less healthy than those of non-Māori and high-income New Zealanders, they experience a greater burden of diet-related disease reflecting their higher average BMI and higher risk of cardiovascular diseases and diabetes [4], and are more responsive to changes in food prices [23].

With any food pricing policy there is a risk of unintended consequences such as shifts from taxed foods to others that are equally or even more unhealthy. Our models suggest that a sodium tax could increase saturated fat purchases (by 3%). Such cross-PE effects could offset positive effects of health-related taxes but in this case the overall effects on population mortality rates remained positive. Consideration of the full implications of tax and subsidy packages is, therefore, critical.

Relative to other strategies to prevent obesity and diet-related disease, health-related food taxes and subsidies are likely to be highly cost-effective. Previous studies found that taxes on unhealthy foods and beverages would be cost-saving and considerably more cost-effective than individually-focussed weight reduction programmes or community or school-based education programmes [52]. Although no panacea, evidence to date suggests that targeted food pricing policies could be an effective component of a multifaceted strategy to tackle New Zealand’s high burden of diet-related disease.

### Conclusion

Health-related food taxes and subsidies could improve diets and reduce mortality from diet-related disease in New Zealand. However, there are many uncertainties in such modelling, not all of which are captured within the reported uncertainty intervals e.g. potential healthier product reformulation by industry in response to taxes and subsidies, which may enhance health gains. Our study adds to the growing evidence base that food taxes and subsidies should improve population health and reduce inequalities, but there is still much room for improvement in the estimation of health impacts.

## Supporting Information

S1 Table New Zealand cross-price food elasticity values. (DOCX)
