# Supplementary material for: Effects of Health-Related Food Taxes and Subsidies on Mortality from Diet-Related Disease in New Zealand: An Econometric-Epidemiologic Modelling Study
Source: PLoS One. 2015 Jul 8;10(7):e0128477. doi: 10.1371/journal.pone.0128477 (PMC4496093; doi:10.1371/journal.pone.0128477)
Supplement: S1 Table — (DOCX) [file pone.0128477.s001.docx]

**Table S1: New Zealand Cross-Price Food Elasticity Values (Means and Standard Errors (SE)), 2006-2010**

|  | Fruit | Vegetables | Beef, Lamb & hogget | Poultry | Pork | Prepared, preserved & processed meat | Fish & seafood | Bread & breakfast cereals | Cakes & biscuits | Pastry cook products | Pasta & other cereal products | Milk, yoghurt & eggs | Cheese & cream | Butter | Margarine & edible oil | Sauces, sugar & condiments | Chocolate, confectionary & snacks | Ice cream | Other grocery food | Non-alcoholic beverages | Carbonated soft drink | Energy drinks | Restaurant food | Ready to eat food |
| --- | --- | --- | --- | --- | --- | --- | --- | --- | --- | --- | --- | --- | --- | --- | --- | --- | --- | --- | --- | --- | --- | --- | --- | --- |
| Fruit | -0.58 (  0.05  ) | 0.06 (  0.03  ) | -0.05 (  0.04  ) | -0.01 (  0.05  ) | 0.04 (  0.06  ) | -0.04 (  0.03  ) | 0.01 (  0.02  ) | 0.06 (  0.04  ) | -0.17 (  0.05  ) | 0.04 (  0.04  ) | -0.05 (  0.02  ) | 0.01 (  0.03  ) | 0.08 (  0.02  ) | 0.05 (  0.05  ) | -0.04 (  0.02  ) | 0.04 (  0.01  ) | 0.01 (  0.03  ) | 0.00 (  0.01  ) | -0.25 (  0.04  ) | 0.00 (  0.01  ) | -0.11 (  0.04  ) | -0.09 (  0.09  ) | 0.05 (  0.03  ) | -0.21 (  0.04  ) |
| Vegetables | 0.05 (  0.02  ) | -0.88 (  0.03  ) | -0.08 (  0.03  ) | -0.04 (  0.03  ) | 0.00 (  0.05  ) | -0.02 (  0.02  ) | 0.03 (  0.02  ) | -0.03 (  0.02  ) | -0.10 (  0.04  ) | -0.02 (  0.03  ) | 0.03 (  0.01  ) | -0.03 (  0.02  ) | 0.01 (  0.02  ) | -0.08 (  0.03  ) | 0.03 (  0.02  ) | 0.06 (  0.01  ) | 0.02 (  0.02  ) | 0.00 (  0.01  ) | -0.17 (  0.04  ) | 0.03 (  0.01  ) | -0.11 (  0.03  ) | 0.03 (  0.05  ) | 0.08 (  0.03  ) | -0.04 (  0.03  ) |
| Beef, lamb & hogget | -0.07 (  0.04  ) | -0.11 (  0.03  ) | -0.93 (  0.06  ) | 0.05 (  0.05  ) | 0.01 (  0.06  ) | -0.01 (  0.03  ) | -0.05 (  0.03  ) | -0.11 (  0.03  ) | -0.12 (  0.04  ) | -0.08 (  0.04  ) | -0.02 (  0.02  ) | -0.03 (  0.03  ) | -0.04 (  0.02  ) | 0.03 (  0.05  ) | -0.02 (  0.03  ) | -0.05 (  0.02  ) | 0.00 (  0.03  ) | -0.02 (  0.01  ) | -0.28 (  0.05  ) | 0.04 (  0.01  ) | -0.03 (  0.08  ) | 0.15 (  0.06  ) | 0.15 (  0.04  ) | 0.05 (  0.05  ) |
| Poultry | -0.19 (  0.06  ) | -0.14 (  0.05  ) | 0.03 (  0.07  ) | -1.70 (  0.09  ) | 0.21 (  0.11  ) | 0.11 (  0.04  ) | -0.02 (  0.04  ) | 0.08 (  0.06  ) | 0.27 (  0.07  ) | 0.16 (  0.06  ) | -0.03 (  0.03  ) | 0.17 (  0.05  ) | 0.02 (  0.03  ) | 0.25 (  0.07  ) | -0.01 (  0.04  ) | -0.01 (  0.02  ) | -0.04 (  0.05  ) | -0.04 (  0.02  ) | -0.20 (  0.07  ) | 0.05 (  0.02  ) | 0.03 (  0.08  ) | 0.21 (  0.11  ) | 0.10 (  0.05  ) | 0.09 (  0.06  ) |
| Pork | -0.13 (  0.10  ) | -0.34 (  0.11  ) | 0.05 (  0.12  ) | 0.43 (  0.14  ) | -4.51 (  0.43  ) | 0.25 (  0.09  ) | -0.23 (  0.09  ) | -0.01 (  0.11  ) | 0.35 (  0.12  ) | -0.02 (  0.11  ) | -0.07 (  0.04  ) | 0.20 (  0.09  ) | 0.03 (  0.06  ) | 0.17 (  0.13  ) | 0.15 (  0.07  ) | -0.04 (  0.04  ) | 0.11 (  0.09  ) | -0.06 (  0.03  ) | 0.04 (  0.12  ) | 0.12 (  0.03  ) | 0.65 (  0.18  ) | 1.95 (  0.25  ) | -0.05 (  0.09  ) | 0.11 (  0.11  ) |
| Prepared, preserved & processed meat | -0.07 (  0.03  ) | -0.07 (  0.03  ) | -0.02 (  0.05  ) | 0.12 (  0.04  ) | 0.10 (  0.05  ) | -1.05 (  0.03  ) | 0.00 (  0.03  ) | -0.09 (  0.03  ) | -0.06 (  0.03  ) | -0.01 (  0.04  ) | 0.05 (  0.02  ) | -0.07 (  0.02  ) | 0.01 (  0.02  ) | 0.11 (  0.04  ) | -0.09 (  0.03  ) | -0.06 (  0.01  ) | -0.09 (  0.03  ) | -0.06 (  0.01  ) | -0.18 (  0.04  ) | -0.01 (  0.01  ) | -0.06 (  0.04  ) | 0.07 (  0.05  ) | 0.17 (  0.04  ) | -0.10 (  0.04  ) |
| Fish & seafood | -0.02 (  0.03  ) | 0.21 (  0.04  ) | -0.16 (  0.06  ) | -0.06 (  0.05  ) | -0.11 (  0.08  ) | 0.12 (  0.04  ) | -1.68 (  0.07  ) | 0.26 (  0.04  ) | 0.03 (  0.03  ) | 0.06 (  0.04  ) | -0.09 (  0.03  ) | 0.24 (  0.04  ) | 0.18 (  0.03  ) | -0.02 (  0.03  ) | 0.05 (  0.02  ) | 0.03 (  0.02  ) | -0.11 (  0.04  ) | -0.04 (  0.02  ) | -0.22 (  0.06  ) | 0.03 (  0.02  ) | -0.23 (  0.05  ) | -1.04 (  0.17  ) | 0.20 (  0.06  ) | -0.05 (  0.06  ) |
| Bread & breakfast cereals | 0.02 (  0.04  ) | 0.01 (  0.03  ) | -0.12 (  0.04  ) | 0.05 (  0.05  ) | 0.08 (  0.07  ) | -0.05 (  0.02  ) | 0.05 (  0.02  ) | -0.73 (  0.04  ) | 0.06 (  0.05  ) | -0.02 (  0.04  ) | 0.01 (  0.02  ) | -0.15 (  0.03  ) | 0.00 (  0.02  ) | 0.02 (  0.05  ) | -0.09 (  0.02  ) | 0.00 (  0.01  ) | 0.07 (  0.03  ) | 0.00 (  0.01  ) | -0.14 (  0.04  ) | 0.02 (  0.01  ) | 0.06 (  0.06  ) | 0.24 (  0.08  ) | 0.01 (  0.03  ) | -0.09 (  0.03  ) |
| Cakes & biscuits | -0.15 (  0.05  ) | -0.10 (  0.05  ) | -0.06 (  0.06  ) | 0.07 (  0.07  ) | 0.29 (  0.11  ) | 0.00 (  0.04  ) | 0.04 (  0.03  ) | -0.05 (  0.06  ) | -0.97 (  0.10  ) | -0.09 (  0.05  ) | 0.01 (  0.02  ) | -0.04 (  0.04  ) | -0.05 (  0.03  ) | -0.08 (  0.09  ) | -0.06 (  0.04  ) | -0.01 (  0.02  ) | 0.03 (  0.04  ) | -0.03 (  0.01  ) | -0.10 (  0.05  ) | -0.01 (  0.01  ) | 0.11 (  0.11  ) | 0.24 (  0.13  ) | -0.03 (  0.04  ) | -0.06 (  0.05  ) |
| Pastry cook products | -0.05 (  0.06  ) | -0.11 (  0.06  ) | -0.20 (  0.09  ) | 0.05 (  0.09  ) | 0.33 (  0.12  ) | -0.12 (  0.06  ) | -0.15 (  0.05  ) | -0.47 (  0.09  ) | 0.43 (  0.11  ) | -1.52 (  0.18  ) | 0.15 (  0.04  ) | -0.40 (  0.08  ) | -0.09 (  0.05  ) | 0.55 (  0.14  ) | -0.26 (  0.06  ) | -0.09 (  0.03  ) | -0.19 (  0.07  ) | -0.06 (  0.02  ) | 0.32 (  0.11  ) | -0.08 (  0.03  ) | -1.90 (  0.28  ) | 1.23 (  0.22  ) | 0.31 (  0.09  ) | 0.32 (  0.08  ) |
| Pasta & other cereal products | -0.05 (  0.04  ) | 0.10 (  0.04  ) | -0.16 (  0.06  ) | 0.28 (  0.06  ) | -0.05 (  0.07  ) | 0.20 (  0.04  ) | 0.08 (  0.05  ) | 0.27 (  0.06  ) | -0.07 (  0.04  ) | 0.12 (  0.05  ) | -1.70 (  0.04  ) | 0.11 (  0.04  ) | 0.02 (  0.03  ) | -0.02 (  0.05  ) | 0.06 (  0.03  ) | -0.06 (  0.03  ) | -0.12 (  0.04  ) | 0.00 (  0.02  ) | 0.10 (  0.06  ) | 0.07 (  0.02  ) | -0.14 (  0.05  ) | -0.36 (  0.09  ) | 0.05 (  0.06  ) | 0.25 (  0.09  ) |
| Milk, yoghurt & eggs | -0.02 (  0.03  ) | -0.03 (  0.03  ) | -0.01 (  0.04  ) | 0.07 (  0.04  ) | -0.01 (  0.05  ) | -0.07 (  0.02  ) | 0.06 (  0.02  ) | -0.14 (  0.02  ) | -0.04 (  0.05  ) | -0.01 (  0.03  ) | -0.01 (  0.01  ) | -0.86 (  0.05  ) | -0.03 (  0.02  ) | 0.07 (  0.07  ) | -0.01 (  0.02  ) | -0.02 (  0.01  ) | 0.04 (  0.03  ) | 0.00 (  0.01  ) | -0.17 (  0.05  ) | 0.05 (  0.01  ) | -0.03 (  0.04  ) | 0.09 (  0.07  ) | 0.09 (  0.03  ) | -0.04 (  0.04  ) |
| Cheese & cream | 0.18 (  0.04  ) | 0.04 (  0.04  ) | -0.07 (  0.05  ) | -0.01 (  0.05  ) | 0.03 (  0.07  ) | 0.12 (  0.03  ) | 0.06 (  0.03  ) | 0.11 (  0.04  ) | -0.10 (  0.04  ) | 0.04 (  0.04  ) | 0.00 (  0.02  ) | 0.00 (  0.03  ) | -1.04 (  0.04  ) | 0.28 (  0.06  ) | -0.10 (  0.03  ) | -0.04 (  0.02  ) | -0.20 (  0.04  ) | -0.02 (  0.01  ) | -0.21 (  0.05  ) | 0.01 (  0.01  ) | 0.04 (  0.05  ) | -0.07 (  0.06  ) | 0.09 (  0.04  ) | -0.22 (  0.05  ) |
| Butter | 0.03 (  0.14  ) | -0.50 (  0.15  ) | -0.34 (  0.16  ) | -0.40 (  0.15  ) | 0.39 (  0.25  ) | 0.13 (  0.10  ) | 0.06 (  0.08  ) | 0.09 (  0.16  ) | -0.35 (  0.20  ) | -0.56 (  0.14  ) | -0.50 (  0.07  ) | -0.03 (  0.09  ) | 0.01 (  0.09  ) | -0.67 (  0.28  ) | 0.50 (  0.07  ) | -0.57 (  0.07  ) | -0.15 (  0.12  ) | -0.09 (  0.03  ) | -0.49 (  0.16  ) | 0.07 (  0.03  ) | 1.01 (  0.32  ) | 0.29 (  0.30  ) | -0.10 (  0.10  ) | -0.05 (  0.10  ) |
| Margarine & edible oil | -0.27 (  0.06  ) | -0.05 (  0.05  ) | 0.02 (  0.08  ) | 0.15 (  0.07  ) | 0.12 (  0.11  ) | 0.00 (  0.05  ) | 0.04 (  0.05  ) | -0.32 (  0.06  ) | -0.27 (  0.07  ) | 0.03 (  0.06  ) | -0.04 (  0.03  ) | 0.00 (  0.05  ) | -0.07 (  0.04  ) | 0.43 (  0.08  ) | -1.04 (  0.07  ) | -0.08 (  0.03  ) | -0.13 (  0.05  ) | 0.01 (  0.02  ) | -0.16 (  0.07  ) | 0.06 (  0.02  ) | -0.47 (  0.10  ) | -0.62 (  0.13  ) | 0.12 (  0.05  ) | 0.04 (  0.06  ) |
| Sauces, sugar & condiments | 0.13 (  0.03  ) | 0.11 (  0.03  ) | -0.18 (  0.04  ) | 0.15 (  0.03  ) | -0.01 (  0.05  ) | -0.01 (  0.02  ) | 0.05 (  0.02  ) | -0.04 (  0.02  ) | -0.15 (  0.03  ) | 0.05 (  0.03  ) | -0.06 (  0.02  ) | 0.16 (  0.03  ) | -0.11 (  0.02  ) | 0.10 (  0.04  ) | 0.05 (  0.02  ) | -1.32 (  0.02  ) | -0.21 (  0.03  ) | -0.04 (  0.01  ) | -0.12 (  0.04  ) | 0.03 (  0.01  ) | 0.01 (  0.03  ) | -0.22 (  0.05  ) | 0.03 (  0.03  ) | 0.06 (  0.04  ) |
| Chocolate, confectionary & snacks | 0.12 (  0.03  ) | 0.02 (  0.03  ) | -0.02 (  0.04  ) | 0.03 (  0.04  ) | 0.18 (  0.06  ) | 0.01 (  0.02  ) | -0.01 (  0.03  ) | 0.21 (  0.05  ) | 0.04 (  0.03  ) | -0.01 (  0.03  ) | 0.04 (  0.02  ) | 0.08 (  0.03  ) | -0.08 (  0.02  ) | 0.12 (  0.04  ) | -0.02 (  0.02  ) | -0.05 (  0.02  ) | -1.27 (  0.04  ) | -0.08 (  0.01  ) | -0.05 (  0.05  ) | -0.08 (  0.02  ) | 0.31 (  0.08  ) | 0.27 (  0.12  ) | 0.09 (  0.04  ) | 0.07 (  0.04  ) |
| Ice cream | 0.19 (  0.03  ) | 0.19 (  0.03  ) | -0.13 (  0.04  ) | 0.55 (  0.07  ) | -0.01 (  0.04  ) | -0.10 (  0.03  ) | -0.05 (  0.03  ) | 0.11 (  0.02  ) | 0.22 (  0.04  ) | -0.11 (  0.03  ) | 0.08 (  0.02  ) | 0.01 (  0.02  ) | -0.03 (  0.02  ) | -0.03 (  0.02  ) | 0.02 (  0.01  ) | 0.01 (  0.01  ) | -0.09 (  0.03  ) | -1.74 (  0.06  ) | 0.24 (  0.06  ) | -0.06 (  0.02  ) | 0.20 (  0.03  ) | -0.47 (  0.07  ) | 0.21 (  0.05  ) | 0.38 (  0.05  ) |
| Other grocery food | -0.09 (  0.02  ) | -0.12 (  0.02  ) | -0.09 (  0.04  ) | -0.08 (  0.03  ) | 0.01 (  0.03  ) | -0.07 (  0.02  ) | 0.00 (  0.02  ) | -0.10 (  0.02  ) | -0.04 (  0.02  ) | -0.04 (  0.04  ) | 0.03 (  0.01  ) | -0.10 (  0.02  ) | -0.01 (  0.01  ) | -0.07 (  0.03  ) | 0.02 (  0.02  ) | 0.03 (  0.01  ) | 0.04 (  0.02  ) | 0.05 (  0.01  ) | -0.38 (  0.04  ) | 0.03 (  0.01  ) | 0.07 (  0.03  ) | -0.12 (  0.06  ) | -0.19 (  0.04  ) | -0.09 (  0.04  ) |
| Non-alcoholic beverages | -0.10 (  0.03  ) | -0.04 (  0.02  ) | 0.00 (  0.03  ) | 0.05 (  0.02  ) | 0.11 (  0.04  ) | 0.02 (  0.02  ) | 0.04 (  0.02  ) | 0.03 (  0.01  ) | -0.17 (  0.04  ) | -0.03 (  0.02  ) | -0.03 (  0.02  ) | 0.11 (  0.02  ) | 0.09 (  0.02  ) | 0.01 (  0.02  ) | -0.02 (  0.01  ) | -0.10 (  0.02  ) | -0.12 (  0.03  ) | -0.01 (  0.01  ) | -0.13 (  0.05  ) | -1.31 (  0.04  ) | 0.26 (  0.06  ) | -0.42 (  0.08  ) | 0.11 (  0.05  ) | -0.25 (  0.05  ) |
| Carbonated soft drinks | -0.14 (  0.08  ) | -0.27 (  0.07  ) | 0.23 (  0.09  ) | 0.59 (  0.13  ) | 0.06 (  0.18  ) | 0.17 (  0.06  ) | -0.14 (  0.05  ) | -0.21 (  0.10  ) | 0.69 (  0.15  ) | -0.25 (  0.09  ) | 0.13 (  0.03  ) | 0.11 (  0.07  ) | -0.02 (  0.05  ) | 0.67 (  0.19  ) | -0.24 (  0.06  ) | -0.02 (  0.03  ) | -0.01 (  0.07  ) | 0.03 (  0.02  ) | 0.15 (  0.08  ) | -0.18 (  0.03  ) | -1.23 (  0.28  ) | 0.77 (  0.34  ) | 0.14 (  0.07  ) | 0.05 (  0.08  ) |
| Energy drinks | -1.14 (  0.19  ) | 0.39 (  0.16  ) | 0.36 (  0.21  ) | 0.18 (  0.24  ) | 1.78 (  0.42  ) | -0.08 (  0.13  ) | -0.23 (  0.11  ) | 0.32 (  0.20  ) | 3.18 (  0.29  ) | -0.06 (  0.25  ) | 0.19 (  0.08  ) | 0.25 (  0.17  ) | -0.40 (  0.10  ) | 0.49 (  0.26  ) | -0.25 (  0.11  ) | -0.35 (  0.08  ) | -0.58 (  0.13  ) | -0.07 (  0.06  ) | 0.31 (  0.24  ) | -0.71 (  0.09  ) | 2.73 (  0.36  ) | -0.31 (  0.60  ) | 0.08 (  0.18  ) | 0.10 (  0.22  ) |
| Restaurant food | 0.08 (  0.01  ) | 0.12 (  0.01  ) | 0.12 (  0.02  ) | 0.02 (  0.02  ) | -0.05 (  0.02  ) | 0.07 (  0.01  ) | 0.05 (  0.02  ) | 0.14 (  0.02  ) | 0.04 (  0.01  ) | 0.04 (  0.02  ) | 0.00 (  0.01  ) | 0.06 (  0.01  ) | 0.04 (  0.01  ) | -0.07 (  0.02  ) | 0.00 (  0.01  ) | -0.03 (  0.01  ) | -0.03 (  0.01  ) | -0.01 (  0.01  ) | 0.02 (  0.04  ) | -0.06 (  0.01  ) | -0.11 (  0.02  ) | 0.13 (  0.60  ) | -0.88 (  0.18  ) | -0.04 (  0.22  ) |
| Ready to eat food | 0.03 (  0.03  ) | 0.06 (  0.02  ) | 0.15 (  0.03  ) | 0.08 (  0.02  ) | 0.13 (  0.03  ) | 0.03 (  0.02  ) | -0.01 (  0.02  ) | 0.11 (  0.03  ) | 0.08 (  0.03  ) | 0.12 (  0.02  ) | 0.10 (  0.02  ) | -0.06 (  0.02  ) | 0.03 (  0.02  ) | 0.02 (  0.01  ) | -0.01 (  0.01  ) | 0.01 (  0.01  ) | 0.00 (  0.02  ) | 0.05 (  0.01  ) | 0.04 (  0.03  ) | -0.08 (  0.01  ) | 0.10 (  0.03  ) | 0.15 (  0.05  ) | -0.09 (  0.03  ) | -0.93 (  0.04  ) |
